# Carbon Sequestration Potential in the Restoration of Highly Eutrophic Shallow Lakes

**DOI:** 10.3390/ijerph19106308

**Published:** 2022-05-23

**Authors:** Andrzej Skwierawski

**Affiliations:** Department of Water Resources and Climatology, University of Warmia and Mazury in Olsztyn, Plac Łódzki 2, 10-719 Olsztyn, Poland; andrzej.skwierawski@uwm.edu.pl

**Keywords:** shallow lakes, bottom sediment, carbon burial rate, carbon sequestration, restoration of water bodies

## Abstract

The primary goal of the study was to determine the quantity of carbon accumulated in shallow fertile water bodies that were restored after a long period of drainage. Massive drainage of mid-field water bodies took place in north-eastern Poland in the 19th century. Of 143 identified drained lakes (each of more than 1 ha before drying) in the Olsztyn Lakeland, 27 have been restored to their original state through natural rewilding processes or recovery projects. From among the variety of drained water bodies, 8 which have been naturally or artificially restored to their original condition 13 to 47 years ago, were the subject of a detailed study on carbon sequestration. The studied water bodies had high productivity, and they were classified as moderately eutrophic to extremely hypertrophic. An analysis of bottom sediments revealed that, after restoration, the examined water bodies have accumulated 275.5 g C m^−2^ a^−1^ on average, which is equivalent to 10.1 Mg ha^−1^ a^−1^ of carbon dioxide (CO_2_) removed from the atmosphere. Results showed that the evaluated water bodies are effective carbon sinks. Most of the lakes drained in the 19th century are wastelands today, and they can be relatively easily restored to their original condition to create additional carbon sequestration sites. Lake restoration seems to be a cost-effective method both for carbon capture (as additional potential capacity as part of carbon dioxide removal (CDR) methods) and to support the sustainable use of agricultural areas. However, this second goal may be limited by the poor ecological status of such facilities.

## 1. Introduction

Despite the implementation of various climate policy measures, anthropogenic emissions of carbon dioxide (CO_2_) continue to increase, and they were estimated at 10.9 ± 0.9 Gt C a^−1^ on average between 2010 and 2019 [1]. Global emissions increase the concentration of atmospheric CO_2_. Carbon dioxide emissions need to be reduced, and new solutions for reducing carbon pollution must be found to effectively combat climate change. Accurate estimates of the global carbon budget are necessary to determine the effectiveness of measures aimed at combating climate change. Until recently, the role of inland water bodies (especially smaller ones) in the carbon cycle was significantly underestimated. Yet they play a very important role as organic carbon sinks [2,3]. They accumulate approximately 300 Tg C a^−1^, of which 42 Tg C a^−1^ is trapped in lakes, 160 Tg C a^−1^ in retention reservoirs, and 96 Tg C a^−1^ in wetlands, despite the fact that aquatic ecosystems constitute less than 2% of Earth’s surface. In turn, oceans cover 71% of the Earth’s surface but capture only 100 Tg C a^−1^ [4]. According to Cole et al. [5], inland waters (flow-through systems) not only transport matter to the seas (as ‘passive pipes’), but also release CO_2_ to the atmosphere (at a rate of 0.75 Pg C a^−1^) and remove carbon from the cycle through permanent accumulation in sediments (at a global rate of 0.23 Pg C a^−1^). Carbon dioxide emissions from lakes have been estimated at 0.07–0.15 Pg C a^−1^ (0.11 Pg C a^−1^ on average) [5]. However, these estimates were made only for large lakes. Alin et al. [6] found that, globally, large lakes produce 250 Tg C a^−1^, release 90 Tg C a^−1^ to the atmosphere, and capture only 7 Tg C a^−1^ in sediments. According to more recent estimates, 2.9 Pg C a^−1^ is transported to inland water bodies, 0.9 Pg C a^−1^ is transported to seas and oceans, and 1.4 Pg C a^−1^ is released to the atmosphere as CO_2_, whereas the remaining 0.6 Pg C a^−1^ is permanently deposited in bottom sediments [7,8]. These values indicate that the global supply of matter to water bodies may be even three times higher than previously thought, and the amount of carbon processed globally in small water bodies can be even 1 Pg a^−1^ higher than previously thought [9].

Increasing the possibilities for carbon accumulation in the bottom sediments of water bodies can potentially be treated as carbon dioxide removal (CDR), a geoengineering method. Carbon can be sequestered in semi-natural conditions or artificially designed systems that are capable of permanently removing carbon from the fast carbon cycle [10]. Effective solutions for removing significant quantities of carbon from the atmosphere have not been developed to date [11]. One of the existing methods involves the establishment of carbon reservoirs by creating favourable conditions for carbon sequestration. This goal can be achieved through afforestation, but the existing solutions are not effective enough to even slow the increase in the concentration of atmospheric CO_2_ [12]. Thus, in addition to forests, soils, and wetlands, water bodies, too, are potent storage reservoirs of carbon. In highly fertile and degraded water bodies, high levels of biological productivity are associated with a poor ecological status. However, the accumulation of organic matter in such water bodies is enhanced, and organic matter is permanently deposited in bottom sediments [13]. This process occurs in both natural water bodies and anthropogenically modified reservoirs.

In stable inland aquatic ecosystems, sediments are covered with fresh deposits, and carbon is transported to the lithosphere and permanently removed from the atmosphere. Carbon deposition in lake sediments is a long-term process that acts as a link between the fast and the slow (geological) carbon cycle [6]. Some matter is circulated in the fast cycle, but excess matter is deposited and permanently stored in bottom sediments. These mechanisms should be taken into account in strategies aiming to mitigate the adverse effects of climate change. Sedimentation processes and carbon deposition should be promoted, and additional carbon sinks should be created wherever possible. The restoration of formerly drained lakes by refilling them can be an effective method for supporting the reduction of the amount of carbon found in the fast carbon cycle—including atmospheric CO_2_.

The aim of this study was to determine the amount of carbon storage in shallow, fertile water bodies. The study was conducted on eight lakes that were restored after about a century of drainage. The hypothesis was that bottom sediments can accumulate a potent stock of carbon. Research on the sediment layer accumulated since the restoration enabled a scientific analysis of the studied lakes. Based on the deposition rate and high organic matter content in bottom sediments, it was assumed that the water bodies can act as efficient carbon sinks on a local scale.

## 2. Materials and Methods

### 2.1. Study Area

**Figure 1:**
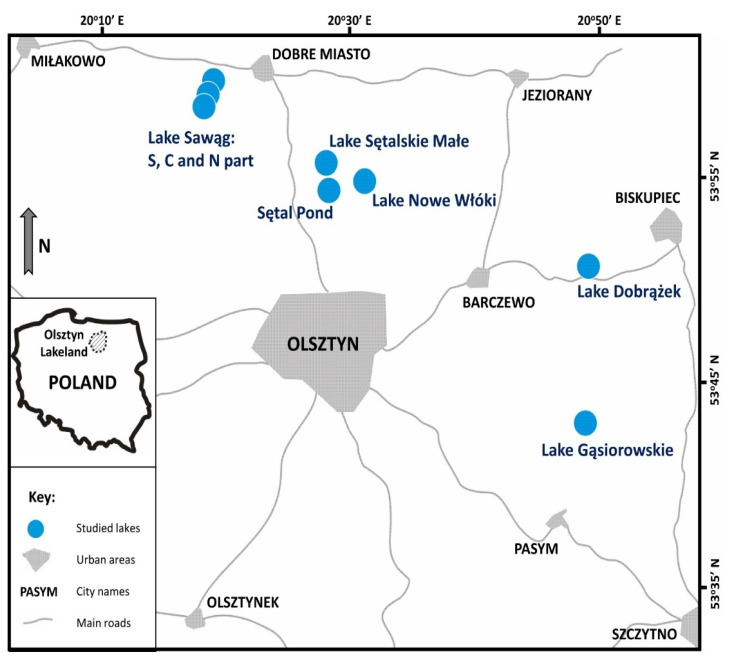
Research area and study sites in the Olsztyn Lakeland.

The study was conducted in the Olsztyn Lakeland, which is part of the Masurian Lakeland geographic region in north-eastern Poland (Figure 1). In geomorphological terms, the Olsztyn Lakeland was formed in the last glacial period, and it is characterised by highly diverse landforms, where differences in elevation range from more than ten to several tens of metres. Elevation ranges from 100–140 m a.s.l. (above sea level) to more than 200 m a.s.l. in terminal moraine landforms. The Olsztyn Lakeland features the highest number of lakes in all mesoregions of the Masurian Lakeland. There are 300 lakes with a combined area of 154.2 km^2^, and they cover 4.04% of the lakeland [14].

The analysed group of shallow water bodies remained dry over a period of around 100 years and have been restored in recent decades. In the 19th century, many lakes were partially or even completely drained to create land for agricultural production [15]. Wetlands were converted to meadows, which were regarded as particularly valuable. The most extensive drainage campaigns were conducted between 1860 and 1900. An analysis of drainage programmes conducted in the Olsztyn Lakeland revealed that 143 water bodies larger than 1 ha had been completely drained. The total drained area was estimated at 3000 ha, which indicates that nearly a third of all water bodies in the region had been eliminated [15]. Of those, since the 1980s, 27 lakes have been restored to their original (or near-original) state through natural rewilding processes or recovery projects.

Eight water bodies that had remained dry for around 100 years were selected for the study. Three of them were separate parts of former Lake Sawąg, which has not been fully restored and presently comprises three independent water bodies: northern (Sawąg N), central (Sawąg C), and southern (Sawąg S). The main features of the studied lakes are presented in Table 1, and, additionally, the description of individual lakes is available in the Supplementary Materials S1.

### 2.2. Methods

The status of the analysed water bodies was determined based on field observations conducted in the growing seasons of 2009 to 2013. Measurements were conducted four times per season. Electrolytic conductivity (EC) and chlorophyll concentration (CHL) were measured with the YSI6600 multiparametric probe (YSI Inc., Yellow Springs, OH, USA), and water transparency was determined using a black-and-white Secchi disk with a diameter of 20 cm. The total phosphorus (TP) and soluble reactive phosphorus (SRP) concentration in water samples taken simultaneously with in situ measurements was determined in a laboratory, with the use of ammonium molybdate and tin(II) chloride in mineralised (TP) and not mineralised (SRP) samples. The trophic status of the evaluated water bodies was classified according to the method proposed by Vollenweider and Kerekes [16]. The method is a probabilistic approach to the determination of trophic status, based on open boundaries between trophic categories (i.e., oligotrophic, mesotrophic, eutrophic, hypertrophic). The final result is determined as the resultant between the states indicated for individual indicators (phosphorus, chlorophyll, and Secchi depth), which makes it possible to determine the dominant trophic status of the lake ecosystem.

Bottom sediments were sampled through ice cover in the central part of each lake, during the winter seasons of 2010–2012, with the use of a Beeker sampler (Eijkelkamp Soil & Water, Giesbeek, The Netherlands) for extracting undisturbed sediment cores. The sediments were sampled using the scatter points method within the open water area of each lake. As a rule, 5–10 soundings were sampled with the probe to obtain representative cores for further analyses. Samples were collected to a depth of 0.7–1.2 m from the sediment surface. Sediments that have accumulated since lake restoration, as well as deeper layers from the dry period before drainage, were clearly distinguishable based on morphological properties in all the lakes. From among a larger number of reclaimed reservoirs tested in the described manner, eight reservoirs were selected as a reference for this specific study—those that met two conditions: (1) the part of sediment accumulated after restoration was identifiable by morphological features (colour, granulometry, structure); (2) the date (year) of the lake reclamation was known.

The sediment cores were divided into 10-cm layers, with the exception of the border between the new and old sediment (sediments after restoration, and meadow soil before the refilling of the lake). Samples in separate 10-cm layers were taken from several (usually 5) cores and were collected as one average sample (this was dictated by the limited funds for sediment analyses).

Immediately after sampling, the physical properties of the collected samples were described, and further analyses were performed in the laboratory of the Department of Water Resources and Climatology of the University of Warmia and Mazury in Olsztyn. Bulk density was determined using the gravimetric method, dividing sediment weight by volume. The water content was determined as the fraction of mass loss relative to the initial mass of a fresh sediment sample after drying at a temperature of 105 °C. Organic matter content was quantified based on the loss of mass after ignition at 550 °C. The amount of carbon was determined based on organic matter content using the average 1.724 factor, as described by Pribyl [17]. Carbonate was determined based on calcium content, which was measured by atomic absorption spectrometry (AAS) in the certified Agricultural Chemistry Laboratory in Olsztyn. Carbon sequestration in bottom sediments was quantified based on the results of analyses and morphometric data, and the accumulation rate of organic matter in bottom sediments was determined separately for each water body. It was calculated using the formulas:

C_seq_ = M_OM_/T_R_·A_L_

M_OM_ = %OM·M_DS_/100·1.724

M_DS_ = %DM·SpD·A_L_·S_L_/100

where:

C_seq_—carbon sequestration [g C m^−2^ a^−1^]
T_R_—time since restoration [y]
A_L_—lake area [m^2^]
M_OM_—mass of organic matter in sediment after restoration [t]
%OM—average organic matter content in sediment [%]
M_DS_—mass of dry sediment [t]
%DM—average percentage of dry matter in sediment [%]
SpD—average specific density of sediment [g cm^−3^]
S_L_—sediment layer thickness after restoration [m].

Additional accumulation in carbonates was calculated similarly, but using calcium concentrations.

## 3. Results

The water quality analysis revealed high levels of eutrophication in the examined water bodies. Lakes Nowe Włóki, Lake Sawąg, and the Sętal Pond had particularly high trophic state indices. Seasonal blooms of cyanobacteria in the study period led to considerable variations in phosphorus and chlorophyll levels (Table 2), in particular between spring (absence of blooms or moderate blooms) and late summer (intense blooms) when Secchi depth decreased to 0.2–0.5 m. Phosphorus concentrations in the Sętal Pond and Lake Sawąg C were three times the critical level of the standard trophic system (total *p* > 100 μg dm^−3^, according to [18]), and these water bodies were classified as hypertrophic (Table 3).

Lakes Gąsiorowskie and Sętalskie Małe were characterised by stable clear-water conditions with a prevalence of vascular plants, which was reflected by low chlorophyll concentrations and the highest Secchi depth (Table 2). These lakes were classified as eutrophic, but the analysed parameters in Lake Gąsiorowskie were close to the values indicative of mesotrophic conditions, whereas the status of Lake Sętalskie Małe tended towards the hypertrophic (Table 3). Lakes Dobrążek and Sawąg N occupied the middle range of values between Lakes Gąsiorowskie and Sętalskie Małe, but the limits for hypertrophy were exceeded in these water bodies, too. Therefore, all of the studied lakes were characterised by a poor ecological status.

Bottom sediments of the studied water bodies were highly similar in terms of physical and chemical properties despite differences in morphometric parameters, catchment characteristics, and trophic status. Unconsolidated sediments with high water content (85.7% on average) and low specific density (1.11 g cm^−3^ on average) were identified in the surface layer (Table 4). Thick layers of highly hydrated (>90%) and unconsolidated sediments are observed in the shallow water bodies that do not dry out [19].

Surface sediment layers deposited after restoration with a thickness of 15–30 cm and high organic matter content: 17.8–45.4% DM (dry matter); 35.7% DM on average, was identified in the studied water bodies (Table 4).

**Figure 2:**
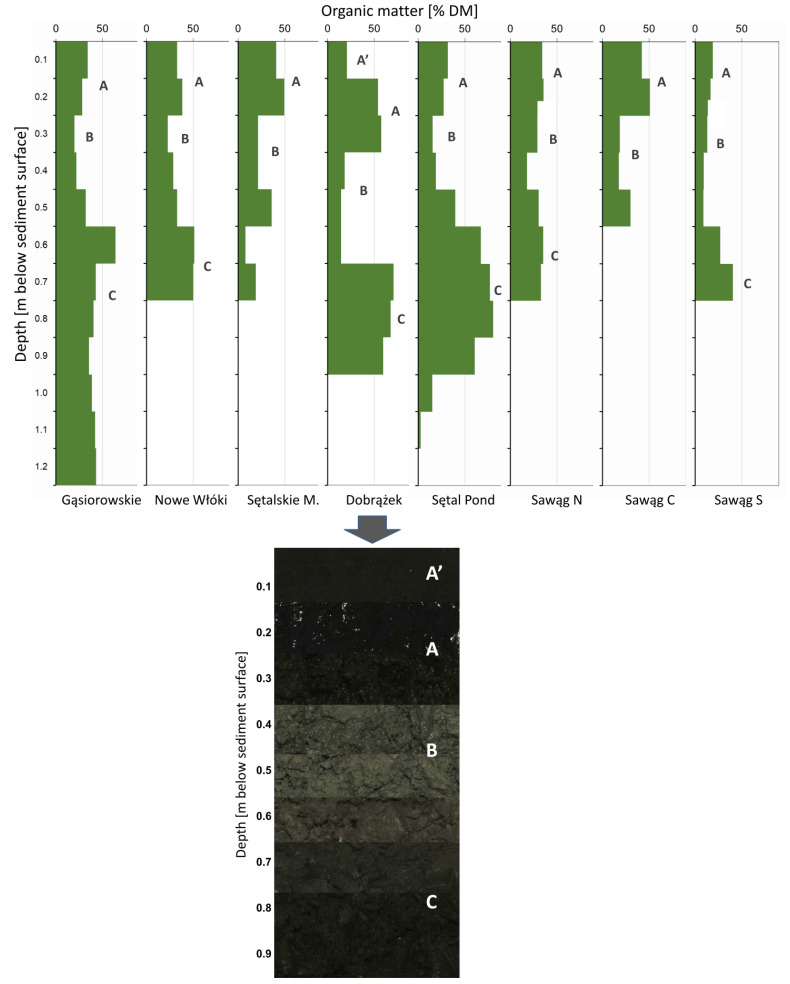
Organic matter distribution in the whole sampled sediment cores in the analysed water bodies, and a visual example of the layers in the core taken from lake Dobrążek; A’—unconsolidated surface sediments; in Lake Dobrążek these were atypical, disturbed by the increased influx of matter from the catchment area (reconstruction of the main route near the lake); A—sediments from after the lake reclamation, with more stabilised lower parts; B—more mineralised sediments (meadow soil) before reclamation, likely influence of the drying period; C—deeper layers with more organic matter from before the lake was drained.

The studied water bodies lay dry over a period of around 100 years. The sediments associated with this period are characterised by their higher specific density and lower organic matter content. The lower organic matter content of deep sediment cores most likely resulted from mineralisation under dry conditions, which led to the release of considerable quantities of CO_2_ into the atmosphere. These layers were more consolidated, and they showed a higher specific density and lower water content (Table 4). These processes occurred in a roughly a 20-cm-thick layer below contemporary sediments (Figure 2). After lake restoration, new material was deposited on the previous sediment surface.

Based on an analysis of sediment cores, the rate at which organic matter accumulated in lakes after restoration was determined as the average from long-term intervals. In polymictic lakes whose natural state has never been disrupted, sediments are strongly resuspended due to intense water mixing or fish activity. As a result, core segments documenting specific short-term periods in a lake’s history cannot be identified. The composition of such sediments is averaged over long-term (multi-annual) cycles. This kind of sediment constituted a well-mixed or, at most, poorly stratified mass, although their lower limit (which determines the time of reclamation) was clearly marked in each lake.

Individual lakes showed a high accumulation of sediment accumulation rate, from 4.3 to 14.3 with the mean value of 8.6 mm a^−1^ (Table 5). The amount of carbon accumulated as organic matter in contemporary layers of sediments ranged from 111 to 405 g C m^−2^ a^−1^ and 264.5 (±101.6) g C m^−2^ a^−1^ on average. The accumulation of organic matter in bottom sediments indicates that a certain quantity of carbon is removed from the fast carbon cycle. The parameters of the older and deeper sediments, located below the zone affected by drying (layers ‘C’ in Figure 2) indicate that carbon sequestration is a long-term process. Excess carbon is continuously removed from the cycle despite the high internal dynamics of polymictic lakes.

An analysis of the profile of sediments sampled from each water body revealed that carbon storage was disrupted by lake drainage (Figure 2). Deep sediment layers were characterised by lower organic matter content, which could indicate that intensified mineralisation led to the release of CO_2_ from the deposits that accumulated during the century-long dry period.

The high organic carbon content of the sediment layers predating drainage demonstrated that the evaluated water bodies had been highly fertile before they were drained in the 19th century (Figure 2). Despite the lakes having remained dry for nearly 100 years, organic carbon has been preserved in deeper sediment layers to this day. The high water table in the moist meadows of empty lake basins stabilised organic matter below the aerated layer. In the studied lakes, these observations were made in layers at an estimated depth of 0.5–0.6 m below the sediment surface.

Bottom sediments also contain calcium carbonate that is precipitated during biological processes. In the analysed sediments, the average content of calcium carbonate was determined at 11.0 g C m^−2^ a^−1^ (Table 5). However, this accounted for only 4% of the carbon present in sediments, compared with the carbon accumulated in organic matter. Calcium carbonate accumulated mostly in contemporary sediments, and it was present in trace amounts in sediments from the dry period.

**Figure 3:**
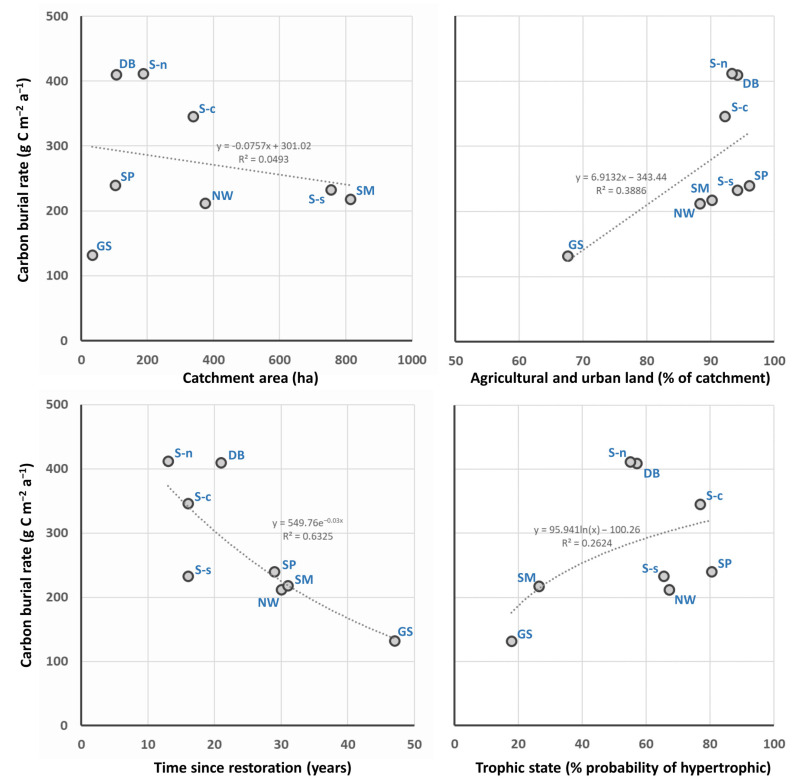
Relationships between indicators characterising the studied lakes and level of matter accumulation in their sediments; Lake denotations: GS—Gąsiorowskie; NW—Nowe Włóki; SM—Sętalskie Małe; DB—Dobrążek; SP—Sętal Pond; S-n—Sawąg (northern part); S-c—Sawąg (central part); S-s—Sawąg (southern part).

In summary, all the studied lakes had a relatively high level of accumulation of matter in bottom sediments compared to typical lake conditions. Based on previous studies of such lakes [20,21,22], it can be hypothesised that, in the conditions of the Olsztyn Lakeland region, the summer periods are warm enough for intensive phytoplankton blooms in conditions of high nutrient abundance, and that, at the same time, the winter periods are cool enough to limit the extent of organic matter mineralisation in sediments. This is indicated by the high seasonal variability of the indicators of primary organic matter production in such lakes. However, levels of matter accumulation differed significantly between lakes. These differences, however, were not closely related to the measured features of the examined lakes (Figure 3). The found correlations were statistically significant only with regard to the relationship between the carbon burial rate and the period from reclamation (correlation coefficient −0.798 significant at *p* = 0.05). This may lead to the conclusion that water bodies accumulate more material in the initial period after restoration, and there is also the possibility that, in the middle term, the matter deposited in the sediment is partially undergoing mineralisation. Old sediment layers (marked as ‘C’ in Figure 2) indicate, however, a permanent accumulation of sediments with a high content of organic matter—even higher than that recorded in modern sediments. Among the studied objects, Gąsiorowskie Lake is an exception in this comparison as it features the most favourable catchment conditions and the lowest trophic state as measured in matter accumulation (Figure 3).

Extrapolation of the data shows the potential for carbon sequestration based on the applied restoration. An analysis of changes occurring in the evaluated water bodies (i.e., lakes that were drained in the 19th century) indicates that, to date, 805 ha of the lakes’ original area of 3000 ha (determined before drainage) have been naturally or artificially restored [15]. Based on these observations, the present rate of CO_2_ equivalent accumulation was determined at 8130 Mg a^−1^, and CO_2_ sequestration could reach 30,000 Mg a^−1^ if the original surface area of the studied water bodies were restored. These estimates apply to only one type of drained lakes described in this study and located in the small geographic mesoregion of the Olsztyn Lakeland (3820 km^2^), but they are similar to the anticipated outcomes of afforestation schemes by the Carbon Forests Programme implemented by the Polish government.

## 4. Discussion

### 4.1. Carbon Accumulation in the Bottom Sediments of Water Bodies

The results of the research indicate that a large amount of organic matter is accumulated in the bottom sediments of hypertrophic lakes. Carbon reaches inland water bodies from external sources, mainly via two routes: with water flowing out of the catchment and by photosynthetic assimilation of atmospheric CO_2_, which constitutes the first step in the biological carbon cycle [5,23]. Matter that is synthesised during primary production and is not remineralised in an aquatic ecosystem is partly eliminated from the carbon cycle and exported outside that ecosystem. In the overall carbon balance, lakes are net producers of CO_2_ (atmospheric emissions exceed absorption), but matter is also accumulated in bottom sediments [24,25].

High carbon sequestration in the sediments of the studied lakes (Table 5) proves the occurrence of such a process of carbon withdrawal, and even if there is no certainty as to the persistence of sediment accumulation in the upper sediment layer, the high concentration of organic matter in the deeper layers of the cores (Figure 2) testifies to the persistence of the accumulation. Eutrophication processes were advanced in the studied lakes. The average rate of accumulation (excess production) was very high, at 275.5 g C m^−2^ a^−1^ (Table 5). The average organic matter content of bottom sediments exceeded 30% (Table 4), which is much higher than in the lakes of the Yangtze River valley (<2%) and in typical sediments of deep, large lakes (0.5 to 6.4%). Similar organic matter content (~25%) was reported in Swedish lakes in the boreal zone [26].

In a study by Dean and Gorham [4], the average rate of accumulation reached 5 g C m^−2^ a^−1^ in large lakes and 72 g C m^−2^ a^−1^ in small water bodies. In the temperate climate (50–60° latitude), primary production in large lakes was determined at 37 g C m^−2^ a^−1^ on average, and 11.7% of matter was accumulated in bottom sediments [6]. In the shallow lakes of Florida, organic matter content was estimated at 30%, and carbon was accumulated at a rate of 63–177 g C m^−2^ a^−1^ [27]. In the shallow eutrophic lakes in the Yangtze River valley (China), the organic matter content of bottom sediments was below 2%. In large but relatively shallow water bodies, high temperature and exposure to wind promote the aerobic mineralisation of sediments. In these lakes, the rate of accumulation varied widely from 5 to 373 g C m^−2^ a^−1^, and carbon deposition in sediments since 1850 was determined at 0.6 to 15.5 kg C m^−2^, with an average of approximately 5 kg C m^−2^ [28].

Lakes are classified as hypertrophic when primary production exceeds 400 g C m^−2^ a^−1^ [16], but much higher values have also been reported in the literature, for example in the hypertrophic Lake Teganuma in Japan, where primary production was determined at 1450 g C m^−2^ a^−1^ [29]. Therefore, the observed accumulation level in the studied lakes, even in the case of maximum values exceeding 400 g C m^−2^ a^−1^ (Table 5), seems to be possible. By comparison, in terrestrial ecosystems such as forests, primary production rarely exceeds 100 g C m^−2^ per annum [30]. However, in aquatic ecosystems, primary production has a cyclical character, especially in polymictic lakes, where matter is recycled in successive cycles and is only partly sedimented.

**Figure 4:**
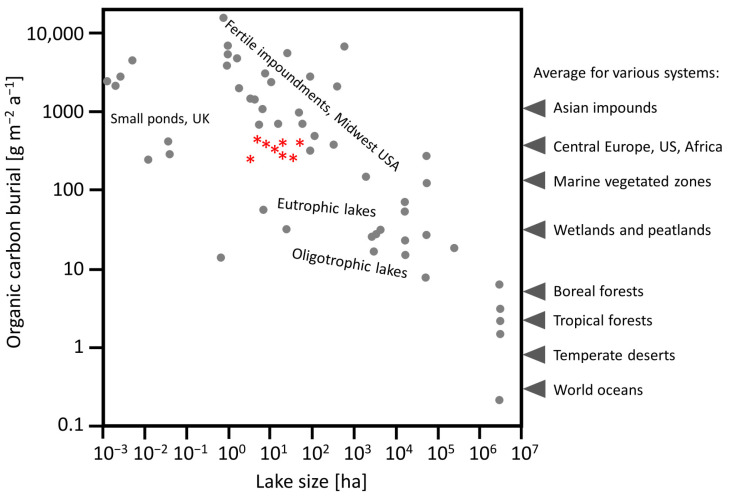
Accumulation of organic carbon in the studied lakes (marked with red asterisks) compared to values observed in various ecosystems (grey circles); comparative data and graph pattern according to Downing [9], modified.

Downing et al. [13] examined 40 lakes and found that sediment organic carbon burial rates were higher than those assumed for fertile retention reservoirs by previous studies, and much higher than those measured in natural lakes, and it was considerably higher in small than large water bodies. Compared to this data, the lakes analysed in this study were in the medium range by area and the accumulation of 264.5 g C m^−2^ a^−1^ (Table 5) was near the low end of the range found by Downing et al. [13] (Figure 4), but this was still significantly more than all the other water body types presented by Downing [9].

Research indicates that organic carbon sequestration, particularly in small water bodies, is much higher than previously thought and is higher than in any other ecosystem type around the world: 10 times higher than in wetlands, 1000 times higher than in boreal forests, and 10,000 times higher than in oceans [9]. According to estimates, 33% of the carbon transported by rivers to seas can be sequestered in ponds. The surface deposition of organic carbon is greater in lakes than in oceans due to higher levels of primary production per unit area in freshwater ecosystems and rapid accumulation of bottom sediments in lakes with a high proportion of unmineralised organic matter [4].

Eutrophication is one of the most widespread environmental problems affecting most lakes in the world. Eutrophication has various consequences, as it influences the biological diversity of lakes as well as their suitability for agricultural production. Eutrophication leads to higher productivity due to the supply of biogenic elements from agricultural catchments. The increase in water temperature induced by climate change also speeds up the circulation of matter in ecosystems and increases both primary production and respiration in eutrophic lakes. The environmental conditions in highly eutrophic and hypertrophic lakes are expected to deteriorate, which will most likely increase carbon sequestration in these water bodies. According to Verspagen et al. [31], a further increase in atmospheric CO_2_ concentrations will intensify phytoplankton blooms in lakes. On the other hand, the sequestration of atmospheric carbon will increase in such ecosystems. Rose et al. [32] found that contemporary sedimentation rates in 71% of the analysed lakes were higher than in the 19th century. Climate change and intensive human activities in catchment areas have contributed to a particularly high increase in the accumulation rate of matter in shallow lowland lakes [33].

High biomass production and its incomplete degradation also contribute to the accumulation of organic matter in the bottom sediments of the analysed lakes (Table 4 and Table 5). Higher temperatures intensify biomass production and affect the availability of carbon. The increase in atmospheric CO_2_ levels can also enhance cyanobacterial blooms, particularly in water bodies with low alkalinity and high nutrient loads [31]. Excess reactive phosphorus, which does not restrict primary production in the early stages of eutrophication, contributes to algal blooms. In the present study, the analysed lakes were also highly abundant in reactive phosphorus (Table 2). Among the factors that contribute to carbon storage are also higher temperatures, which increase the intensity of the respiration and methanogenesis processes, which in turn may result in increased emissions of CO_2_ and CH_4_ to the atmosphere.

Dry lake beds also exert a negative impact on the carbon cycle. Lakes that are temporarily, periodically, or permanently drained contribute to CO_2_ emissions to the atmosphere. According to Marcé et al. [34], drained lakes can contribute as much as 0.22 Pg C a^−1^ to the global carbon balance, which is equivalent to more than 10% of CO_2_ emissions from terrestrial regions. Data obtained from sampled cores (Figure 2; Table 5) suggests that these processes occurred also in studied lakes during the drying period of around one century.

### 4.2. Prospects for the Restoration of Formerly Drained Water Bodies

Most research studies investigating the environmental significance of water bodies fail to recognise their role as carbon traps. Organic matter is effectively sequestered in small lakes and artificial reservoirs but, due to their small size, these functions are not acknowledged and are considered only on the local scale or on the scale of catchments [35,36]. Aquatic ecosystems accumulate high amounts of carbon globally; therefore, ponds and small lakes could play a considerable role as organic carbon sinks in the biosphere [9]. Until recently, small inland water bodies were regarded as sites that contribute to water retention and biological diversity on the local scale only [15], whereas their role in global processes was largely disregarded [9]. Most research focuses on large lakes, whereas in quantitative terms, smaller water bodies constitute the predominant type of aquatic ecosystems around the world [37].

Water bodies are also landscape elements that retain water and decrease the risk of drought; therefore, their role in mitigating the adverse consequences of global warming cannot be overlooked. Analyses that focus only on the trophic status of water bodies could suggest that restoration measures are not justified because they do not increase water bodies’ suitability for economic activity. Due to adverse morphometric parameters and the high susceptibility to degradation (which also depends on catchment basin parameters), restored lakes are characterised by high or moderate trophic levels, and their suitability for agriculture and fisheries is limited. These observations were confirmed by the present findings. However, previously dewatered lakes could be restored due both to their hydrological benefits and to their ability to capture carbon. Lakes such as those described in this study can be restored: fully—by recreating the entire lake basin (27 such sites have been successfully restored in the Olsztyn Lakeland); or partly—the outflow is dammed lower to create wetlands (61 such sites have been created, and most were restored spontaneously when drainage works were abandoned) [15]. Partly restored water bodies (wetlands) most likely accumulate large amounts of carbon from the biomass produced by vascular plants. However, carbon accumulation in these wetlands should be considered as a potential source of methane release. Further research, including quantitative analyses, is needed to determine carbon sequestration and methane emissions from restored lakes.

Drained lakes should be restored, and new water bodies can be created not only because they effectively accumulate carbon, but also because they deliver a variety of environmental benefits. The main characteristics of fully restored lakes include the following:

- Water bodies are dominant landscape features that enhance the local scenery;
- Water bodies are habitats for aquatic fauna and flora (this depends on their ecological state), which contribute to the biological diversity of rural areas;
- Measures aiming to improve water quality are difficult to implement in moderately to highly eutrophic water bodies. Restored lakes are initially characterised by clear-water conditions, but the clear state is much more difficult to stabilise than the turbid state with increased phytoplankton growth [38]. For this reason, the rationale behind many lake restoration projects is often questioned;
- Restored lakes can accumulate significant amounts of carbon in bottom sediments, and they can make some contribution to carbon sequestration possibilities, as a method that does not require large technical and financial outlays; also, carbon sequestration in restored lakes (Table 5) can be higher, even up to 20 times, than in meadows in the temperate climatic zone, according to the literature [39];
- Lakes can be used for economic activities (as water intakes), recreational purposes (depending on their trophic status), and fisheries;
- Lake restoration projects can activate local communities. The importance of water bodies that serve many functions is recognised by local residents. Deteriorating lakes deplete local resources, which increases the awareness of environmental issues.

This study indicates that restored lakes contribute to the protection of water resources, enhance the local (mainly agricultural) landscape, and broaden ecosystem services. However, the maintenance of good ecological status in lakes poses a considerable challenge. The restored lakes in this study are shallow and susceptible to degradation. It should also be noted that the lakes in north-eastern Poland are generally characterised by a turbid water state, which partially limits the range of their potential functions [21,22]. In such water bodies, strict protective measures are needed to improve water quality. Effective management of effluents in the catchment area poses the greatest problem because even small point sources of pollution can significantly compromise a lake’s ecological status. Restored lakes are also highly susceptible to non-point-source pollution due to the absence of natural biogeochemical barriers such as littoral vegetation, which takes more than 10 years to fully develop. For this reason, a protective zone where commercial activities are banned should be planned around restored lakes to capture pollutants from the direct catchment.

In many lakes that were drained in the past, damming structures can easily be built due to the presence of open channels and underground drainage pipes [15]. Lakes Nowe Włóki, Sętalskie Małe and Dobrążek, as well as Lake Sawąg, the largest of the analysed water bodies, are good examples of the above. These lakes were restored by raising the outflow or by building simple damming structures in the outlet channel. However, there are also additional costs associated with the restoration of former lakes. First, is a cost of land taken by a water reservoir, however, nowadays there are mostly marginal lands, with low productivity, or abandoned since the introduction of mechanization in agriculture; such land requires large expenditures for the maintenance of land reclamation, and the presence of soft subsoils (lake sediments), makes it difficult to carry out agricultural works [15]. There is also environmental cost of wetland-type former lakes restoration, because it may be associated with the loss of valuable wetland ecosystems. The functioning of wetland-type former lakes requires more detailed exploratory research. The biggest barrier to the spread of reclamation seems to be a land ownership, especially where it is currently used for agriculture.

Lake restoration benefits should also be analysed from a broader perspective. Recent decades have witnessed a catastrophic decline in biological diversity [40,41,42,43]. New habitats should be created to mitigate this risk, but very few sites are available in modern built-up areas. Lake restoration creates new opportunities for increasing habitat diversity and combating the adverse consequences of climate change. In restored lakes characterised by high biological productivity, excess atmospheric CO_2_ is captured by aquatic vegetation and accumulated due to rapid eutrophication and intense phytoplankton blooms, processes that are regarded as adverse from the utilitarian (lake management) point of view.

## 5. Conclusions

The results of this study indicate that the restoration of drained lakes and, by analogy, the creation of new water bodies can be effective in carbon sequestration. Sediment accumulation rates and the amount of organic matter accumulated in contemporary sediments suggest that the studied water bodies play a significant role as carbon sinks. An analysis of eight lakes revealed that, after restoration, they can accumulate 275.5 g C m ^2^ a^−1^ on average, which is equivalent to 10.1 Mg ha^−1^ a^−1^ of CO_2_ removed from the atmosphere. The study showed that the restored water bodies will accumulate more carbon in the initial period soon after filling up than in the later period after their condition stabilises.

The restoration of drained lakes shows a variety of benefits for nature and human: it promotes natural small water retention and increases the area of aquatic habitats, though the promotion of biological diversity is significantly limited due to the poor trophic status. Simultaneously, by acting as carbon traps, the lakes can reduce CO_2_ emissions by as much as 30,000 Mg a^−1^ when fully restored.

New terrestrial carbon sinks need to be identified to improve the carbon balance. Due to their high productivity, shallow water bodies of the temperate climate zone could play an important role in this process. Favourable conditions for carbon accumulation may be related to the climatic characteristics of the region, i.e., warm summers, which are conducive to biomass production, and cold winter periods, which are conducive to the accumulation of matter. However, this is only a hypothesis and requires additional research, especially in the context of the impact of climate change on the size and stability of carbon accumulation in bottom sediments.

The example of the analysed lakes indicates that carbon sinks can easily be created in areas with sufficient water to fill up drained depressions and maintain stable water levels. Lake restoration seems a cost-effective method for: (1) carbon capturing, as an alternative to technical CDR methods, and (2) supporting the sustainable use of agricultural areas. However, the second goal may be limited by the poor ecological status of such facilities.

## Figures and Tables

**Table 1:** Location and characteristics of the analysed water bodies (based on our own study).

Water Body	Location	Area		Agricultural Land in the Catchment	Maximum Depth	Time since Restoration
		Lake	Catchment (Including Direct Catchment)			
		ha		%	m	Years
Gąsiorowskie	53°43′13″ N 20°48′53″ E	6.9	34	68	3.5	47
Sętalskie Małe	53°54′41″ N 20°28′49″ E	12.5	813 (115)	90	2.6	31
Dobrążek	53°49′50″ N 20°47′40″ E	9.3	105	94	2.5	21
Nowe Włóki	53°53′58″ N 20°31′42″ E	19.8	375	88	2.7	30
Sętal Pond	53°54′14″ N 20°28′57″ E	3.7	103	96	1.6	29
Sawąg N	53°59′04″ N 20°19′44″ E	53.2	187	93	4.0	13
Sawąg C	53°58′36″ N 20°18′51″ E	15.4	338 (151)	92	3.5	16
Sawąg S	53°58′15″ N 20°18′46″ E	33.4	755 (417)	94	3.5	16

**Table 2:** Indicators of trophic status of analysed water bodies measured in growing seasons (April–October) of 2009–2013: mean values and standard deviation (abbreviations: EC—specific electrolytic conductivity; SD—Secchi depth; CHL—chlorophyll concentration; TP—total phosphorus concentration; SRP—soluble reactive phosphorus concentration).

Water Body	EC [μS cm^−1^]	SD [m]	CHL [μg dm^−3^]	TP [μg dm^−3^]	SRP [μg dm^−3^]
Gąsiorowskie	389 (±17)	1.8 (±0.3)	8.9 (±4.2)	79 (±60)	8 (±5)
Sętalskie Małe	305 (±37)	1.4 (±0.3)	14.9 (±12.1)	130 (±207)	16 (±10)
Dobrążek	347 (±37)	1.0 (±0.4)	22.0 (±10.3)	138 (±90)	20 (±13)
Nowe Włóki	284 (±35)	0.7 (±0.3)	26.8 (±12.4)	190 (±119)	14 (±8)
Sętal Pond	229 (±29)	0.6 (±0.3)	51.6 (±50.6)	346 (±242)	50 (±99)
Sawąg N	390 (±48)	1.0 (±0.3)	21.6 (±11.3)	233 (±126)	54 (±50)
Sawąg C	394 (±38)	1.0 (±0.2)	34.8 (±16.5)	313 (±205)	81 (±75)
Sawąg S	372 (±61)	0.9 (±0.3)	25.6 (±11.6)	247 (±164)	72 (±61)

**Table 3:** Trophic status of analysed water bodies based on concentrations of phosphorus and chlorophyll, and Secchi depth, expressed as probability of a given trophic class, based on the method described by Vollenweider and Kerekes [16].

Water Body	Probability of Trophic State [%]			
	Oligotrophic	Mesotrophic	Eutrophic	Hypertrophic
Gąsiorowskie	1.8	31.4	49.0	17.8
Sętalskie Małe	1.0	23.5	49.2	26.4
Dobrążek	0.0	3.4	39.7	57.0
Nowe Włóki	0.0	2.0	30.8	67.2
Sętal Pond	0.0	0.2	19.4	80.5
Sawąg N	0.0	5.4	39.7	55.0
Sawąg C	0.0	0.0	23.2	76.9
Sawąg S	0.0	2.5	32.2	65.4

**Table 4:** Parameters of bottom sediments in the analysed water bodies, calculated as mean values for two vertical zones of sediment cores: ‘After refilling’ means the part of sediment deposited after restoration and ‘Before drainage’ refers to the lower part of sediment, which was influenced by the dry conditions prevailing during the drying period in each water body.

Water Body	Specific Density g cm^−3^		Water Content [%]		Organic Matter [% DM]		Calcium Content [% DM]	
	After Refilling	Before Drainage	After Refilling	Before Drainage	After Refilling	Before Drainage	After Refilling	Before Drainage
Gąsiorowskie	1.07	1.18	86.6	71.2	31.2	21.0	11.43	2.02
Sętalskie Małe	1.10	1.29	88.5	65.6	45.4	21.5	1.15	0.10
Dobrążek	1.06	1.29	89.6	54.9	44.3	15.8	1.02	0.01
Nowe Włóki	1.13	1.27	87.5	67.1	35.7	24.7	5.72	0.23
Sętal Pond	1.15	1.37	82.4	56.5	29.6	17.4	0.30	0.09
Sawąg N	1.25	1.57	74.7	41.9	17.8	10.4	3.04	0.09
Sawąg C	1.10	1.36	87.7	63.1	46.7	18.3	0.34	0.08
Sawąg S	1.05	1.30	88.7	61.0	35.1	23.5	2.15	0.13
Average	1.11	1.33	85.7	60.2	35.7	19.1	3.14	0.34

**Table 5:** Estimated total and average annual carbon accumulation capacity for the post-restoration period in the analysed water bodies based on available data.

Water Body	Accumulation Rate	Mass of Carbon Buried in the Top Sediment Layer		Carbon Burial Rate		CO_2_ Equivalent	
		in Organic Matter	in CaCO_3_	in Organic Matter	in CaCO_3_	in Organic Matter	in CaCO_3_
	mm a^−1^	Mg		g C m^−2^ a^−1^		Mg ha^−1^ a^−1^	
Gąsiorowskie	4.3	358	68	111	21	4.1	0.8
Sętalskie Małe	6.5	833	11	215	3	7.9	0.1
Dobrążek	14.3	791	9	405	5	14.8	0.2
Nowe Włóki	6.7	1163	96	196	16	7.2	0.6
Sętal Pond	6.9	257	1	239	1	8.8	0.1
Sawąg N	11.5	2619	231	379	33	13.9	1.2
Sawąg C	9.4	1842	7	345	1	12.6	0.1
Sawąg S	9.4	557	18	226	7	8.3	0.3
Average	8.6			264.5	11.0	9.7	0.4

## Data Availability

The data presented in this study are available on request from the corresponding author.

## Supplementary Materials

The following supporting information can be downloaded at: https://www.mdpi.com/article/10.3390/ijerph19106308/s1, Supplementary Materials S1: Description of individual lakes, and Figure S1: Photographs of the examined water bodies.
