# Supplementary material for: Carbon Sequestration Potential in the Restoration of Highly Eutrophic Shallow Lakes
Source: Int J Environ Res Public Health. 2022 May 23;19(10):6308. doi: 10.3390/ijerph19106308 (PMC9140842; doi:10.3390/ijerph19106308)
Supplement: Supplementary file 1 [file ijerph-19-06308-s001.zip › ijerph-1705558-supplementary.pdf]

Carbon Sequestration Potential in Restoration of Highly Eutrophic Shallow Lakes

Andrzej Skwierawski

S1: Supplementary materials: description of individual lakes

Lake Gąsiorowskie is an elongated water body with poorly developed shoreline. With the neighboring Lakes Giławskie and Krzywe it belongs to a system of anthropogenically modified water bodies. Water levels were originally much higher, and Lake Gąsiorowskie was more extensively connected with the other water bodies. Lake management programs significantly decreased water level at the beginning of the 20th century, and the basin of Lake Gąsiorowskie was utilized as a waterlogged meadow. In the early 1960s, water level was raised, and the lake was permanently restored. At present, the lake is characterized by clear water conditions, and it is significantly colonized by aquatic vegetation with floating leaves (*Nuphar lutea, Nymphaea alba, Potamogeton natans*) and poorly developed coastal vegetation communities (mainly *Phragmites australis* and *Carex sp.*). The lake's catchment has an area of only 34 ha, and it features farmland and forests. Built-up land along the northern shore of the lake accounts for 12% of the catchment's area. The lake is used for recreational purposes, and there are numerous resorts and summer cottages in the developed part of the catchment (Figure 1S).

Lake Nowe Włóki was restored around the year 1980 by building a weir at the outlet. The Refilling of the lake has been carried out, because the existing meadows were no longer suitable for agricultural use due to excessive waterlogging and too high costs of rebuilding the drainage system. The lake has poor vegetation, with a narrow strip of *Phragmites australis* and *Carex sp.* along the shore and small patches of *Potamogeton natans* on the water table. The lake's catchment has a total area of 375 ha. Arable land occupies more than 60% of the catchment's area with no major biogeochemical barriers separating the land from the lake. Built-up areas are located along the northern shore of the lake. Meadows and pastures occupy 25% of the catchment's area. Previous research demonstrated that Nowe Włóki is a eutrophic lake with considerable phytoplankton growth and seasonal blooms of cyanobacteria (Skwierawski 2006).

Lake Sętalskie Małe was restored together with Lake Nowe Włóki in 1980. Lake surface is dominated by macrophytes and overgrown with dense patches of *Phragmites australis*. Only 40% of the lake's area (5.3 ha) is free of vegetation. The bottom of the lake is covered by submerged vegetation, mostly *Ceratophyllum demersum*. The lake has a permanent clear-water status despite inflows from the adjacent Lake Sętalskie Duże which carry large amounts of phytoplankton as well as similar concentrations of biogenic elements in both lakes. The lake's catchment has a total area of 815 ha, and the direct catchment spans 115 ha. The direct catchment is used mainly as arable land which stretches nearly to the shoreline in the northern and western part. After many years of fallowing, arable land was reclaimed in 2005, but it was converted to non-farming purposes in subsequent years.

Lake Dobrążek was restored in the late 1980s. In the past, the lake had been drained via open ditches intersecting the lake basin. The lake is sparsely covered with vegetation, and *Typha latifolia, Phragmites australis* and *Glyceria maxima* mainly grow on a fairly narrow strip of rushes. *Elodea canadensis* also appears in the coastal zone of the lake's bottom. The lake's catchment is used mainly as arable land, excluding on the southern peninsula which is occupied by permanent grasslands. A large farm occupies the western shore of the lake. The southern part of the catchment is intersected by a busy road. The road was reconstructed in 2009-2011, which induced significant changes in hydrological conditions in the catchment. The lake was also a popular fishing site.

Lake Sawąg is composed of three separate water bodies situated at an altitude of 109.0 (northern part: Sawąg N) and 109.5 m a.s.l (both southern: Sawąg S and central: Sawąg C part). Originally, the lake had an area of around 200 ha, and water level was much higher. According to historical sources, Lake Sawąg was drained in 1870-1871 at the request of a local landowner. The lake was restored gradually between 1994 and 2000. Its most recent northern part was formed accidentally by a ruptured drainage pipe. Lake Sawąg has an agricultural catchment. All parts of the lake are fishing sites. The lake

is characterized by high fertility and cyanobacterial blooms in summer, mostly in the central part (Skwierawski, 2012). The lake has narrow, but quite varied stripes of rush vegetation, with a predominance of species such as: *Phragmites australis*, *Phalaris arudinacea*, *Typha latifolia* and *Alisma plantago-aquatica*. Aquatic vegetation is sparse due to poor water clarity and the lake's short existence.

The Sętal Pond is a small water body with a small island in the southern part of the pond is colonized by waterfowl. The rush vegetation is mainly composed of *Typha latifolia* and *Equisetum fluviatile*. The catchment has an area is used mainly for agricultural purposes. The catchment is also occupied by the northern part of the Sętal village with a population of 357. The southern part of the catchment features steeply sloping terrain which is used as arable land. Farmland stretches nearly to the shoreline, and the pond is strongly exposed to non-point source pollution. Strong fishing pressure has induced changes in the shoreline. In the group of the analyzed water bodies, the Sętal Pond is most susceptible to cyanobacterial blooms in summer. Blooms occur each year, and they are highly intense in some seasons.

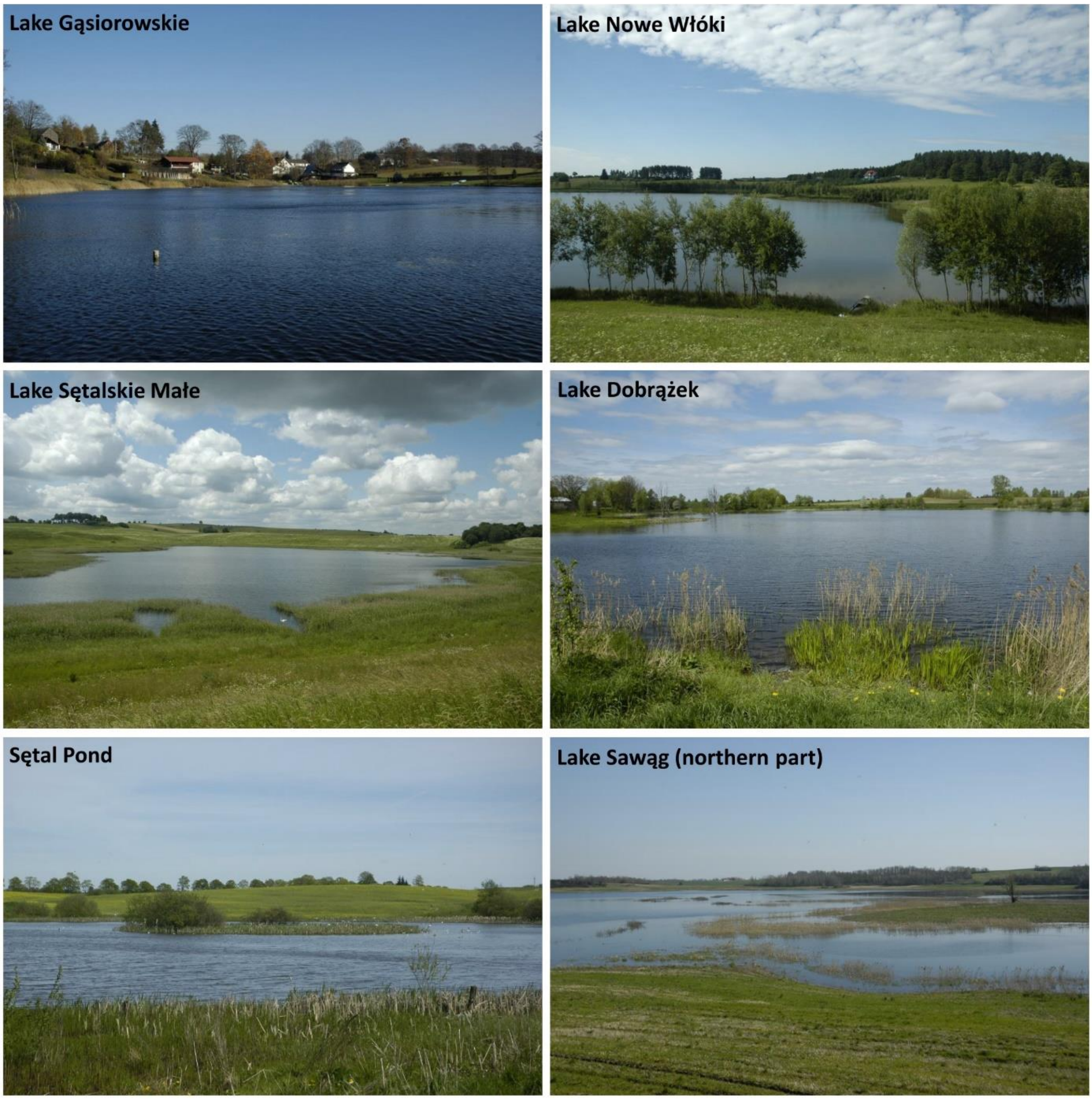

Figure S1. Photographs of the examined water bodies during the spring season (photo by author)
